# Establishment and validation of a nomogram for coronary artery lesions in children with Kawasaki disease

**DOI:** 10.3389/fcvm.2024.1522473

**Published:** 2025-01-14

**Authors:** Chong Hu, Xiao Yan, Henglian Song, Qin Dong, Changying Yi, Jianzhi Li, Xin Lv

**Affiliations:** ^1^ Clinical Laboratory, Children's Hospital Affiliated to Shandong University, Jinan, China; ^2^ Clinical Laboratory, Jinan Children’s Hospital, Jinan, China; ^3^ Department of Hematology, Qingdao Municipal Hospital, Qingdao, China; ^4^ Department of Hematology and Oncology, Jining No. 2 People’s Hospital, Jining, Shandong, China

**Keywords:** Kawasaki disease, nomogram, LASSO, coronary artery lesions, predictors

## Abstract

**Background:**

The nomogram is a powerful and robust tool in disease risk prediction that summarizes complex variables into a visual model that is interpretable with a quantified risk probability. In the current study, a nomogram was developed to predict the occurrence of coronary artery lesions (CALs) among patients with Kawasaki disease (KD). This is especially valuable in the early identification of the risk of CALs, which will lead to proper diagnosis and treatment to reduce their associated complications.

**Methods:**

Retrospective clinical data of 677 children diagnosed with KD who were treated in the Children's Hospital Affiliated with Shandong University were analyzed. All the participants were divided into the CAL group and no CAL group according to their coronary echocardiography results. Least absolute shrinkage and selection operator (LASSO) regression was applied for the identification of the most informative predictors of CAL. Based on this, a nomogram was developed for accurate risk estimation.

**Results:**

The data were divided into a training set and a validation set. Receiver operating characteristic analysis, calibration curves, and decision curve analysis all supported the high accuracy and clinical utility of this model. LASSO regression highlighted five key predictors: sodium, hemoglobin, platelet count, D-dimer, and cystatin C. A nomogram based on these predictors was established and successfully validated in both datasets. In the training set, the AUC was 0.819 and in the validation set it was 0.844. The C-index of the calibration curve in the training set was 0.820, while in the validation set it was 0.844. In the decision curve analysis, the predictive benefit of the model was greater than zero when the threshold probability was below 95% in the training set and below 92% in the validation set.

**Conclusion:**

The predictive factors identified through the LASSO regression approach and the development of the nomogram are important contributions in this respect. This model had a high predictive accuracy and reliability for identifying high-risk children in the very early stage of disease with remarkable precision, laying the foundation for personalized treatment strategies and targeted treatment and providing a strong scientific basis for precise therapeutic intervention.

## Introduction

Kawasaki disease (KD) is a benign but self-limited systemic vasculitis, primarily affecting children. The clinical presentation is very variable in children with recurrent fever, rash, and diffuse congestion of conjunctiva involving lips and bilateral bulbar conjunctivas (1, 2). If not diagnosed and properly treated at an early stage, serious complications include the development of coronary artery lesions (CALs), which is one of the most important cardiovascular outcomes of the disease (3). Recent studies have stated that cardiac complications due to KD now rank among the top causes of acquired heart disease in children (4, 5). Thus, the early and precise evaluation of the risk of a CAL in the clinical process is very important for reducing the complications related to cardiovascular events and improving prognosis and also enables children to receive more active and timely treatment.

An extended range of laboratory indicators was used in our study, so it was useful for defining the best predictive factors related to the risk of the development of a CAL. Considering its advantages, least absolute shrinkage and selection operator (LASSO) regression showed better performance when compared with other techniques based on conventional statistics. Thus, as compared with such classic variants, LASSO regression generates simpler models, can avoid overfitting issues, and can better solve the multicollinearity issues that arise between the independent variable features, leading to the generation of high-quality, precise models, and enhancing reliability (6). A nomogram, a new, increasingly used tool in the realm of risk prediction, is able to combine heterogeneous elements of clinical symptoms, demographic information, and laboratory data into a succinct and intuitive graphical format. It graphically illustrates the contribution of each variable to the predicted outcome and thus allows clinicians to avoid complex regression analysis. Using scores corresponding to individual variables, physicians can easily predict risk probabilities (7, 8). Unlike most of the traditional prediction models, which are usually overcomplicated or difficult to interpret, a nomogram simplifies the process and increases the practical utility of risk assessments. This makes it especially relevant for early disease risk prediction and the implementation of precision medicine strategies (9). Our study focuses on aiding physicians in predicting the likelihood of CAL development during the baseline period of KD, specifically at initial diagnosis and before the initiation of drug therapy. By analyzing variations in laboratory indicators with high predictive value, we aim to facilitate early interventions that improve clinical outcomes. In addition, our findings provide a theoretical framework for future monitoring and management strategies for Kawasaki disease.

## Materials and methods

### Data collection

This was a retrospective study using the clinical data of children diagnosed with Kawasaki disease at Children's Hospital affiliated with Shandong University from January 2020 to December 2023.

Analysis was conducted, including demographic and clinical data, initial laboratory characteristics, and echocardiographic reports. Laboratory characteristics mainly included: alanine transaminase (ALT), blood potassium (K), white blood cell count (WBC), sodium (Na), red blood cell distribution width-SD/CV (RDW-SD/CV), aspartate aminotransferase (AST), D-dimer, hemoglobin (Hb), platelet count (PLT), albumin (ALB), absolute monocyte count (MONO#), cystatin C (CysC), and AST to ALT ratio (AST/ALT). This study obtained approval from the Ethics Committee of Children's Hospital affiliated with Shandong University.

### Inclusion and exclusion criteria

According to the standards issued by the American Heart Association in 2017 (10), the inclusion criteria were as follows: (1) Initial definitive diagnosis of KD. (2) Complete initial laboratory parameters. (3) Complete initial auxiliary examinations such as echocardiography. (4) Responsive to intravenous gamma globulin (IVIG) treatment upon admission. The exclusion criteria were as follows: (1) Those not definitively diagnosed with KD. (2) Individuals with missing initial laboratories and echocardiographic reports. (3) IVIG-resistant patients. (4) Patients who received IVIG treatment at other healthcare facilities. (5) Individuals with a history of Kawasaki disease recurrence. (6) Cases with incomplete clinical records upon admission or termination of treatment due to various reasons. (7) Abnormal laboratory characteristics caused by other diseases. (8) Diagnosed with other congenital diseases, genetic metabolic diseases, rare diseases, and so on.

### Participants and group assignment

**Figure 1:**
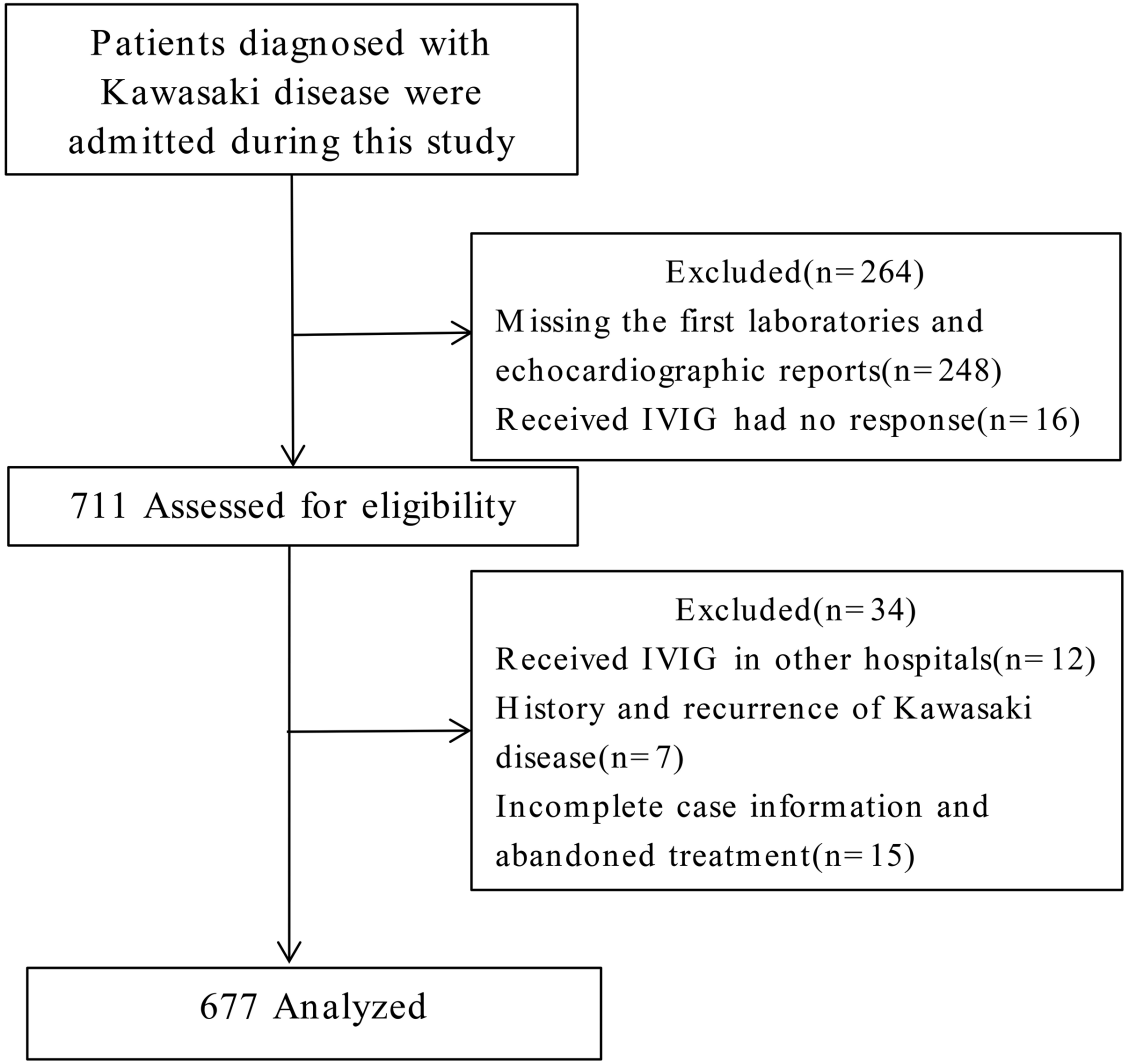
Flowchart of the participants included in the study. IVIG, intravenous gamma globulin.

In this study, 975 patients with KD were diagnosed and treated at our hospital. Based on the inclusion and exclusion criteria outlined above, 248 cases were excluded due to missing initial laboratory and echocardiographic reports, and 16 cases were excluded because of IVIG resistance. Of the remaining 711 patients assessed for eligibility, 34 were further excluded. These included 12 patients who had received IVIG treatment elsewhere, 7 with recurrent KD, and 15 with incomplete clinical records or discontinuation of treatment for various reasons. Ultimately, a total of 677 cases were included in the analysis (Figure 1). A CAL refers to a spectrum of abnormalities in the morphology, structure, and function of coronary arteries in KD. These changes result from systemic vasculitis affecting the coronary arteries during the disease's progression. According to the American Heart Association guidelines published in 2017, echocardiography results are deemed positive (indicating a CAL) if any of the following criteria are met: (1) Z score of the left anterior descending coronary artery or right coronary artery ≥2.5; (2) presence of a coronary artery aneurysm; (3) presence of ≥3 additional suggestive features, such as reduced left ventricular function, mitral regurgitation, pericardial effusion, or *Z* scores of 2.0–2.5 in the left anterior descending coronary artery or right coronary artery. Based on this evaluation method, 205 cases were categorized into the CAL group, and 472 cases into the no CAL (N-CAL) group. To validate the predictive model's performance, the total cohort was divided into a training set (473 cases) and a validation set (204 cases) in a 7:3 ratio.

### Statistical analysis

Statistical analysis was performed by SPSS software (version 28.0) and R software (version 4.3.0). The continuous variables that had a normal distribution were expressed as mean ± SD (standard deviation), while variables that had a skewed distribution were expressed as median (interquartile range). The categorical variable was age (divided into ≤ 1, 1–5, and ≥ 5 years). Group comparisons were made using the *t*-test, Mann–Whitney *U*-test and Chi-square test. LASSO regression was conducted using R software (version 4.3.0) with 10-fold cross-validation to select predictive variables. Based on the LASSO regression results, logistic regression analysis screened the optimal predictive characteristics for a CAL, and then a nomogram, calibration curve, receiver operating characteristic (ROC) curve, and decision curve analysis (DCA) were employed to verify the model's calibration, discrimination, and clinical effectiveness. *P* < 0.05 was considered statistically significant.

## Results

### Laboratory data of participants

Of the 677 cases included in this study, 205 cases were categorized into the CAL group and 472 cases in the N-CAL group based on coronary echocardiography. The analysis results showed statistically significant differences in RDW-SD, Na, Hb, PLT, D-dimer, CysC, and AST/ALT (*P* < 0.05) between the groups. There were no significant differences for the other laboratory markers (*P* > 0.05) (Table 1). There was no statistically significant difference between the training and validation sets (*P* > 0.05), as shown in (Table 2).

**Table 1:** Baseline characteristics of all the included patients.

Characteristic	KD-CAL (*n* = 205)	KD-N-CAL (*n* = 472)	Statistic	*P*
Sex, *n* (%)				
Female	73 (35.61)	182 (38.56)	χ^2^ = 0.53	0.467
Male	132 (64.39)	290 (61.44)		
Age (years), *n* (%)				
≤1	55 (26.83)	122 (25.85)	χ^2^ = 3.21	0.201
≥5	16 (7.80)	59 (12.50)		
1–5	134 (65.37)	291 (61.65)		
ALT (U/L)	21.44 ± 8.84	21.67 ± 25.83	*t* = −0.17	0.866
K (mmol/L)	4.78 ± 0.44	4.73 ± 8.72	*t* = 0.09	0.928
RDW-SD (%)	40.13 ± 3.43	39.39 ± 3.96	*t* = 2.48	0.014
WBC (×10^9/^L)	7.49 (5.63–9.37)	7.01 (5.72–8.57)	*Z* = −0.95	0.342
Na (mmol/L)	141.00 (137.00–146.00)	148.00 (147.00–149.00)	*Z* = −12.35	<0.001
AST (U/L)	38.00 (31.00–45.00)	36.00 (31.00–43.00)	*Z* = −1.47	0.141
Hb (g/L)	102.00 (97.00–106.00)	123.00 (117.00–128.00)	*Z* = −16.26	<0.001
PLT (×10^9/^L)	559.00 (489.00–654.00)	292.00 (251.75–345.25)	*Z* = −18.02	<0.001
D-dimer (mg/L)	1.36 (0.99–2.18)	0.45 (0.26–0.88)	*Z* = −12.79	<0.001
ALB (g/L)	42.30 (39.40–47.50)	41.40 (39.00–44.23)	*Z* = −1.92	0.055
MONO# (×10^9/^L)	0.36 (0.26–0.56)	0.40 (0.32–0.53)	*Z* = −1.35	0.177
CysC (mg/L)	1.32 (1.10–1.66)	0.77 (0.58–0.89)	*Z* = −15.61	<0.001
RDW-CV (%)	13.20 (11.90–14.90)	13.00 (12.20–14.10)	*Z* = −0.69	0.492
AST/ALT	2.00 (1.36–2.67)	2.22 (1.51–3.26)	*Z* = −2.97	0.003

ALT, alanine transaminase; K, blood potassium; WBC, white blood cell count; Na, blood sodium; RDW-SD/CV, red blood cell distribution width-SD/CV; AST, aspartate aminotransferase; Hb, hemoglobin; PLT, platelet count; ALB, albumin; MONO#, absolute monocyte count; CysC, cystatin C; AST/ALT, AST to ALT ratio.

**Table 2:** Comparison of laboratory markers between the training and validation sets.

Laboratory marker	KD-training set (*n* = 473)	KD-validation set (*n* = 204)	Statistic	*P*
ALT (U/L)	21.70 ± 23.55	21.38 ± 18.36	*t* = 0.173	0.863
K (mmol/L)	4.91 ± 8.70	4.36 ± 0.54	*t* = 0.911	0.362
RDW-SD (%)	39.59 ± 3.87	39.66 ± 3.73	*t* = −0.208	0.835
WBC (×10^9/^L)	7.11 (5.69–8.83)	6.98 (5.73–8.75)	*Z* = 0.400	0.689
Na (mmol/L)	148.00 (141.00–149.00)	147.00 (141.00–149.00)	*Z* = 1.316	0.190
AST (U/L)	37.00 (31.00–43.00)	37.00 (31.00–45.00)	*Z* = 0.471	0.638
Hb (g/L)	120.00 (104.00–126.00)	119.50 (103.75–126.00)	*Z* = 0.348	0.728
PLT (×10^9/^L)	327.00 (270.00–468.00)	344.00 (270.00–542.00)	*Z* = 1.318	0.187
D-dimer (mg/L)	0.64 (0.32–1.30)	0.62 (0.32–1.44)	*Z* = 0.648	0.517
ALB (g/L)	41.80 (39.20–45.20)	41.45 (38.50–44.52)	*Z* = 1.278	0.201
MONO# (×10^9/^L)	0.39 (0.31–0.54)	0.39 (0.32–0.52)	*Z* = 0.544	0.587
CysC (mg/L)	0.84 (0.64–1.16)	0.85 (0.69–1.21)	*Z* = 0.835	0.404
RDW-CV (%)	13.10 (12.20–14.30)	13.00 (12.30–14.30)	*Z* = 0.265	0.791
AST/ALT (U/L)	2.12 (1.48–3.00)	2.13 (1.40–3.00)	*Z* = 0.157	0.875

ALT, alanine transaminase; K, blood potassium; WBC, white blood cell count; Na, blood sodium; RDW-SD/CV, red blood cell distribution width-SD/CV; AST, aspartate aminotransferase; Hb, hemoglobin; PLT, platelet count; ALB, albumin; MONO#, absolute monocyte count; CysC, cystatin C; AST/ALT, AST to ALT ratio.

### Potential risk factors indicated by LASSO regression

**Figure 2:**
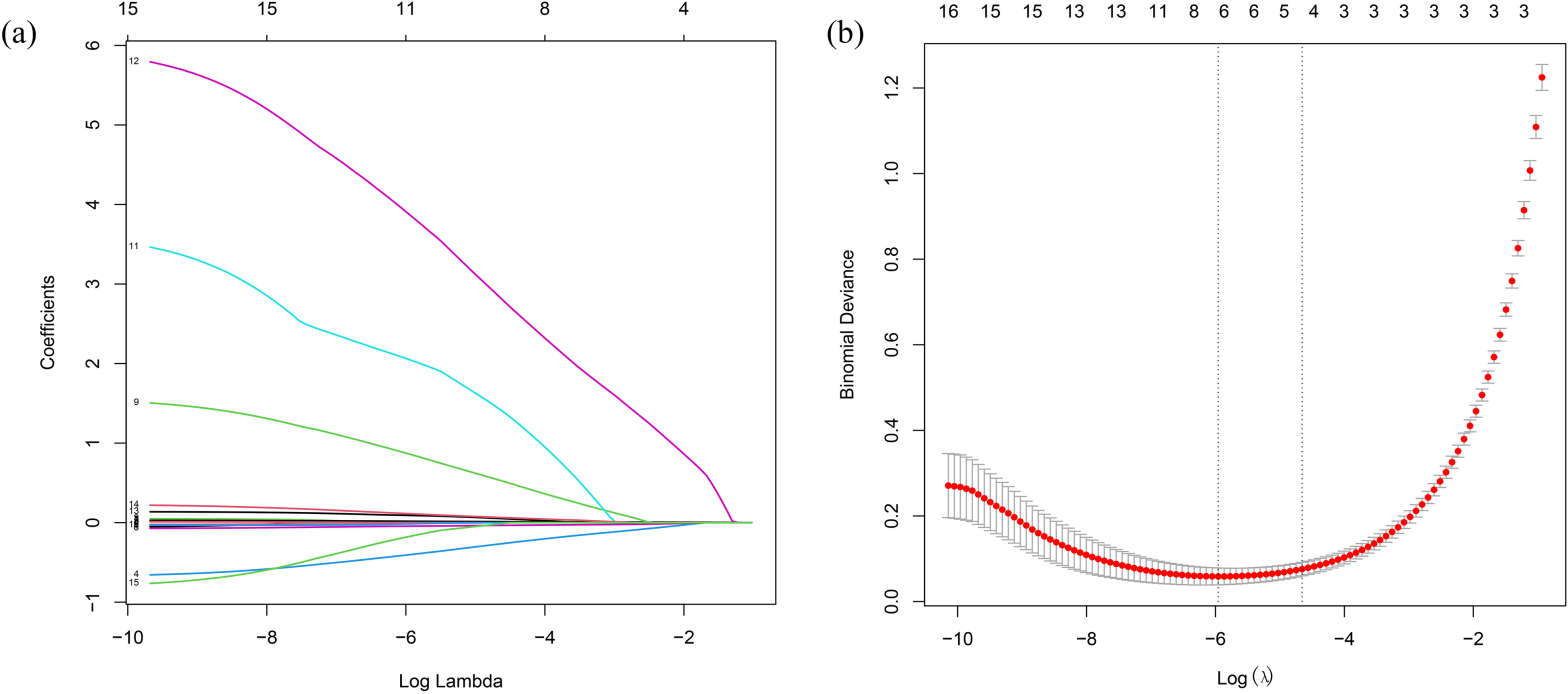
Laboratory marker selection using LASSO regression. **(a)** Optimal parameter (lambda) selection in the LASSO model with 10-fold cross-validation via the minimum criterion. **(b)** LASSO coefficient profiles for the relevant features. LASSO, least absolute shrinkage and selection operator.

LASSO regression analysis was performed on all characteristics with a CAL as the outcome. Based on this analysis (Figures 2a,b), five optimal predictive factors were finally identified: Na, Hb, PLT, D-dimer, and CysC.

### Logistic regression analysis of predictive factors for CAL

These five optimal predictive factors were used in the logistic regression analysis, as shown in Table 3. The results are as follows: Na [odds ratio (OR): 0.83, 95% confidence interval (CI): 0.78–0.89], Hb (OR: 0.96, 95% CI: 0.93–0.99), PLT (OR: 1.01, 95% CI: 1.01–1.01), D-dimer (OR: 2.06, 95% CI: 1.39–3.04), and CysC (OR: 15.75, 95% CI: 5.74–43.21).

**Table 3:** Multivariate logistic regression analysis of risk factors for CALs.

Variable	Beta	SE	OR	95% CI	*P*
Na	−0.18	0.03	0.83	0.78–0.89	<0.001
Hb	−0.04	0.02	0.96	0.93–0.99	0.007
PLT	0.01	0.00	1.01	1.01–1.01	<0.001
D-dimer	0.72	0.20	2.06	1.39–3.04	<0.001
CysC	2.76	0.51	15.75	5.74–43.21	<0.001

OR, odds ratio; CI, confidence interval; Na, blood sodium; Hb, hemoglobin; PLT, platelet count; CysC, cystatin C.

### Establishment of a prediction nomogram model for a CAL

**Figure 3:**
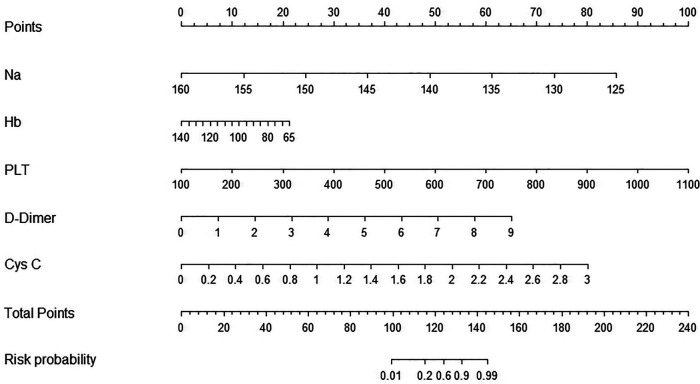
Nomogram model developed for CALs in children with Kawasaki disease.

The five optimal predictive factors were incorporated into the nomogram model (Figure 3). The model provided scores for each variable, which were summed to calculate a total score. This total score corresponded to a line segment's length, allowing for the estimation of the risk probability of a CAL occurrence. The figure illustrates that factors such as hyponatremia, low hemoglobin levels, thrombocytosis, elevated D-dimer levels, and elevated cystatin C levels yielded higher scores, significantly increasing the risk of a CAL.

### Validation and clinical effectiveness of the nomogram model's performance

**Figure 4:**
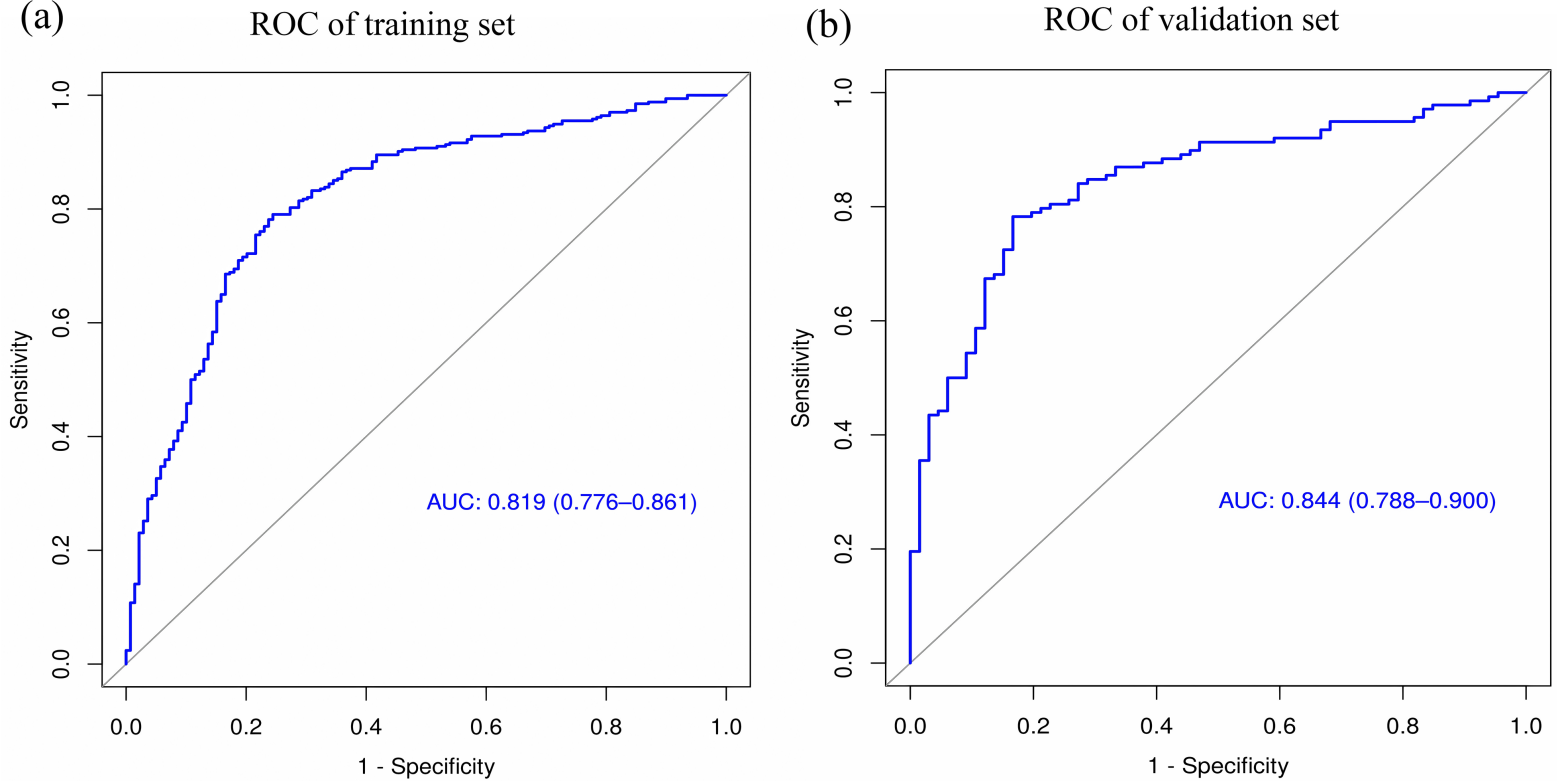
ROC curve analysis of the nomogram model. **(a)** ROC of the training set. **(b)** ROC of the validation set.

**Figure 5:**
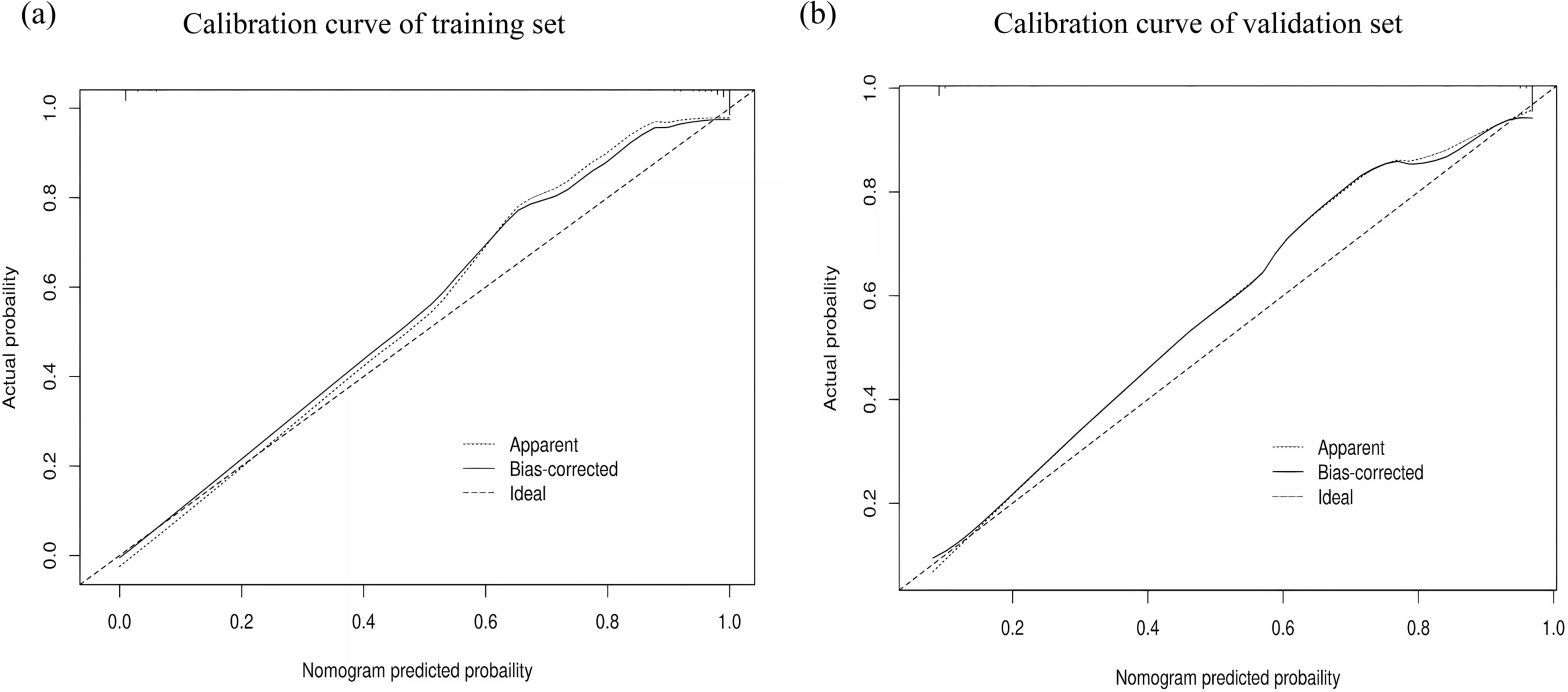
Calibration curve analysis of the nomogram model. **(a)** Calibration curve of the training set. **(b)** Calibration curve of the validation set.

**Figure 6:**
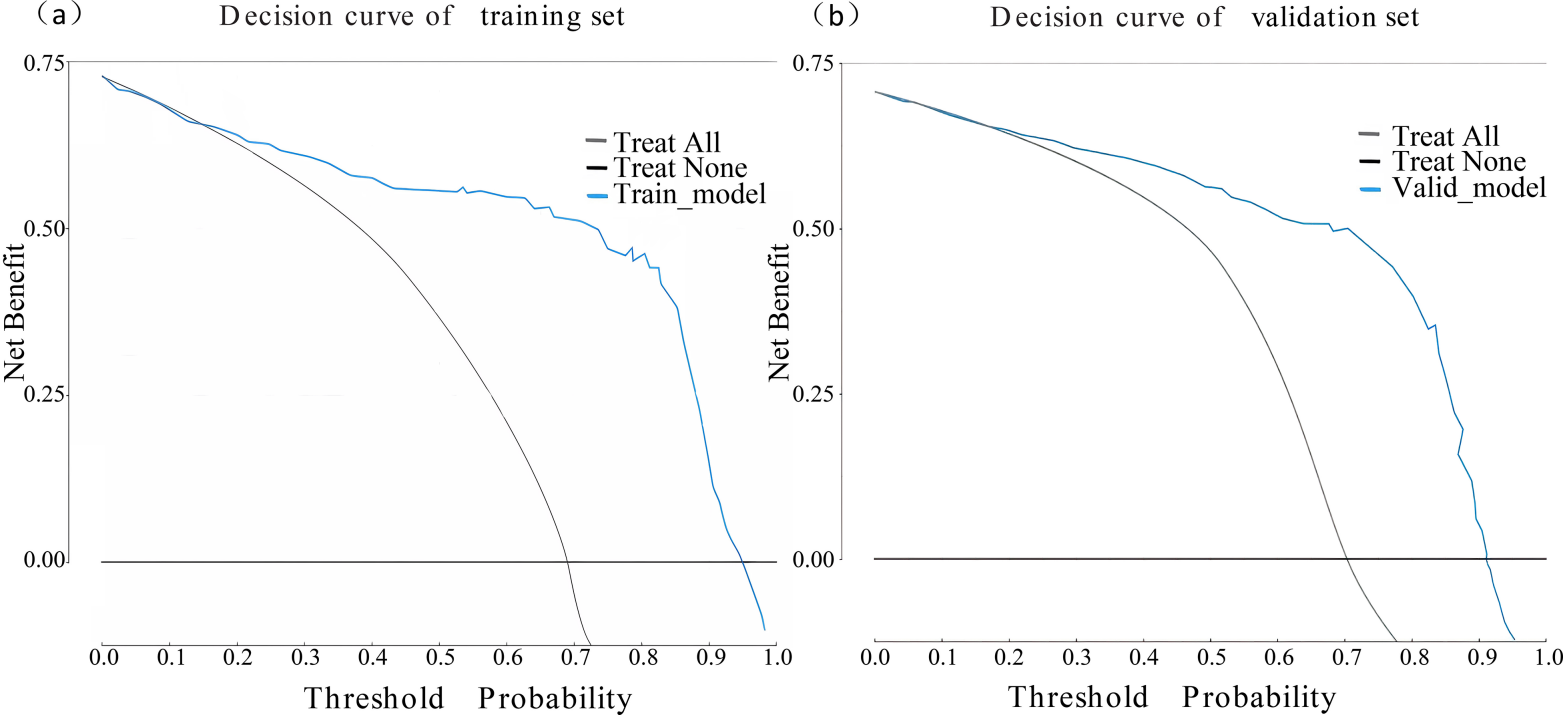
Decision curve analysis of the nomogram model. **(a)** Decision curve of the training set. **(b)** Decision curve of the validation set.

The performance of the nomogram was evaluated using several metrics. ROC analysis demonstrated an area under the curve (AUC) of 0.819 (95% CI: 0.776–0.861) for the training set and an AUC of 0.844 (95% CI: 0.788–0.900) for the validation set (Figures 4a,b), indicating strong predictive efficacy for a CAL. The calibration curve analysis (Figures 5a,b) showed a good fit between the calibration curve and the ideal curve. On these plots, the *X*-axis represents the predicted probability of a CAL, while the *Y*-axis represents the actual probability of a CAL. The diagonal dotted line indicates perfect prediction by an ideal model, while the solid line represents the validation results of the nomogram. A closer alignment of the solid line with the diagonal dotted line reflects better predictive accuracy, demonstrating good consistency between predicted and actual values. The C-index of the calibration curve was 0.820 for the training set and 0.844 for the validation set, supporting the model's strong predictive performance. The DCA (Figures 6a,b) further evaluated the model's clinical utility. In these plots, the *X*-axis denotes the threshold probability, and the *Y*-axis denotes the net benefit. The black horizontal solid line represents the assumption of no disease in all children, the gray solid line indicates the net benefit, and the blue solid line illustrates the decision curve. The analysis revealed that when the threshold probability was below 95% in the training set and below 92% in the validation set, the net benefit of using the model to predict a CAL exceeded zero. This indicates that the model has good clinical effectiveness and practical value in predicting a CAL.

## Discussion

IVIG therapy is the primary treatment for KD. It strengthens the immune response in patients with KD, suppresses the progression of systemic vasculitis, alleviates or halts vasculitic reactions, reduces the occurrence of CALs, and prevents the formation of coronary artery thrombosis (11–13). However, studies have shown that approximately 15%–20% of patients exhibit resistance to initial IVIG treatment, significantly increasing their risk of developing CALs (14–16). Despite this, predicting CAL risk among IVIG-sensitive patients remains crucial. In certain KD cases, whether in the acute stage or diagnosed later, the inflammatory response may have already caused damage to the coronary arteries, even in patients who respond well to IVIG.

Although the exact etiology of KD remains unclear, it is thought to involve abnormal immune system activation in genetically predisposed individuals following microbial infection or environmental exposure. This abnormal activation ultimately leads to acute systemic vasculitis syndrome (17, 18). Our study identified five key predictors, namely, sodium, hemoglobin, platelet count, D-dimer, and cystatin C, that collectively form a novel indicator system for predicting CALs in children with KD. Previous research suggests potential mechanisms for these high-risk factors. Patients with severe hyponatremia often experience intensified inflammation, characterized by prolonged fever and elevated levels of various inflammatory biomarkers. Thus, severe hyponatremia in KD patients may reflect greater disease severity, potentially accelerating the development of a CAL (19, 20). Although not fully explained, the mechanism underlying the decrease in hemoglobin levels among patients with CALs has been claimed to be associated with the increased production of certain cytokines, including interleukin 6 (IL-6) and IL-1β (21). Some studies have reported that changes in red blood cell (RBC) structure and function might independently or in combination affect blood circulation and potentially lead to vascular occlusion (22). Furthermore, early RBC aging and subsequent removal from the circulation may contribute to an increased risk of CALs (23). Thrombocytosis, especially in the acute phase, is a common finding in patients with KD and may contribute to cardiovascular complications in the disease. Recent studies indicate that thrombocytosis in KD results from enhanced platelet stimulation and disturbed apoptosis (24). The sharp increase in PLT as an independent risk factor for CALs is in agreement with previous reports (25). Thus, serial monitoring of PLT in KD patients is an important predictor for the development of a CAL. D-dimer levels reflecting endothelial injury are also an important index. High levels of D-dimer support the relationship between endothelial injury and the risk factors for CAL (26). CysC was a novel predictive marker in this work. While traditionally considered an early biomarker of renal impairment, there is an increasing body of evidence pointing to its strong association with cardiovascular diseases. High levels of CysC might be associated with inflammatory status and increased vascular inflammation, and further cause injury to cardiovascular tissues. CysC is an important molecule in the pathogenesis and development of KD, acting as a good predictor of the presence, severity, and prognosis of a CAL (27, 28).

In this study, LASSO-logistic multivariate analysis was performed using a set of 17 indicators, including demographic, clinical, and laboratory characteristics. LASSO regression has some unique advantages in the management of high-dimensional data and variable selection, where redundant factors are eliminated and the focus is on key predictors. This greatly enhances the accuracy and stability of the prediction model. The nomogram is important in the practical management of Kawasaki disease. It combines early clinical manifestations, laboratory parameters, and other relevant data that are used in disease diagnosis into one nomogram. With systematic analysis and model training, it gives a quantified risk score for suspected KD patients. This tool will be very useful in the early phase of the disease when symptoms are not distinct and helps the clinician in making more accurate assessments about the likelihood of KD. It greatly improves diagnostic precision by reducing the chances of misdiagnosis or missed diagnosis (29, 30). In treatment decision-making, the risk probabilities provided by the nomogram enable physicians to design personalized treatment plans. For example, for patients identified as high-risk for a CAL, the early initiation of high-dose intravenous immunoglobulin shock therapy can be prioritized, while patients categorized as low-risk may be managed with milder approaches. Moreover, during treatment, the nomogram can guide a revision of the therapeutic strategy based on dynamic changes, such as the recovery of body temperature or the speed of decline of inflammatory markers. This facilitates decisions on escalation, adjustment of medication, or maintaining the current treatment strategy for optimal therapeutic effect while reducing unnecessary drug adverse reactions (31, 32). In the prognosis assessment and long-term management, the nomogram plays an indispensable and irreplaceable role. It integrates multiple dimensions of patient information, including residual symptoms after treatment, the degree of recovery of the coronary artery as shown by echocardiography, and whether there is an abnormal inflammatory marker, to predict the possibility of future adverse cardiovascular events. This enables the provision of follow-up intervals appropriate for the level of risk. Shorter follow-up intervals can facilitate early detection and timely management of potential complications in high-risk patients. In contrast, the follow-up interval can be extended for low-risk patients, which may optimize medical resources without affecting the health of patients (8, 33). Clinical cases should be systematically divided into a training set and a validation set to guarantee the reliability and clinical utility of the prediction model. Advanced analytical methods, including ROC curve analysis, calibration curve assessment, and decision curve analysis, were applied to comprehensively evaluate the model's performance. The strictness of this validation process confirms that the model is reliable for practical applications and thus forms a firm basis for using the model in routine clinical practice.

Our study focused on the critical issue of CALs in children with KD, combining an in-depth analysis with the development of a practical prediction tool capable of providing accurate guidance for clinical practices. The research moves away from the traditionally unidirectional methodologies to adopt a multidimensional outlook, hence providing novel insights into the prevention and management of CALs in KD children.

## Conclusion

Hyponatremia, low hemoglobin levels, elevated platelet counts, increased D-dimer levels, and higher cystatin C levels were identified as independent high-risk factors for CALs. The nomogram developed using these factors exhibits strong predictive accuracy, providing an effective tool for estimating CAL risk in patients with KD. This assists physicians in the early identification of high-risk individuals and supports targeted treatment approaches, contributing to the optimal allocation of medical resources.

## Limitation

This research is a single-center study, which introduces certain limitations. The absence of some clinical data and constraints on sample size have precluded mechanistic investigations into the risk factors for IVIG resistance. In the future, we aim to conduct multi-center studies to refine and expand the prediction model framework. In addition, as this study is confined to a single center, there is a slightly higher representation of patients with typical complications compared to other institutions. This may introduce bias in patient distribution, which we anticipate resolving through participation in multi-center collaborative research initiatives.

## Data Availability

The original contributions presented in the study are included in the article/Supplementary Material, further inquiries can be directed to the corresponding authors.
